# Human Platelet Antigen Alleles in 998 Taiwanese Blood Donors Determined by Sequence-Specific Primer Polymerase Chain Reaction

**DOI:** 10.1155/2013/973789

**Published:** 2013-06-20

**Authors:** Shun-Chung Pai, Thierry Burnouf, Jen-Wei Chen, Liang-In Lin

**Affiliations:** ^1^Department of Clinical Laboratory Sciences and Medical Biotechnology, College of Medicine, National Taiwan University, No. 1 Chang-Te Street, Taipei 10048, Taiwan; ^2^Taiwan Blood Services Foundation, 3rd Floor No. 3 Nan-Hai Road, Taipei 10066, Taiwan; ^3^Institute of Biomedical Materials and Tissue Engineering, College of Oral Medicine, Taipei Medical University, Taipei 11031, Taiwan; ^4^Human Protein Process Sciences (HPPS), 59000 Lille, France; ^5^Department of Laboratory Medicine, National Taiwan University Hospital, No. 7 Chung-Shan Southern Road, Taipei 10016, Taiwan

## Abstract

Polymorphism of human platelet antigens (HPAs) leads to alloimmunizations and immune-mediated platelet disorders including fetal-neonatal alloimmune thrombocytopenia (FNAIT), posttransfusion purpura (PTP), and platelet transfusion refractoriness (PTR). HPA typing and knowledge of antigen frequency in a population are important in particular for the provision of HPA-matched blood components for patients with PTR. We have performed allele genotyping for HPA-1 through -6 and -15 among 998 platelet donors from 6 blood centers in Taiwan using sequence-specific primer polymerase chain reaction. The HPA allele frequency was 99.55, and 0.45% for HPA-1a and -1b; 96.49, and 3.51% for HPA-2a and -2b; 55.81, and 44.19% for HPA-3a and -3b; 99.75, and 0.25% for HPA-4a and -4b; 98.50, and 1.50% for HPA-5a and -5b; 97.75 and 2.25% for HPA-6a and -6b; 53.71 and 46.29% for HPA-15a and -15b. HPA-15b and HPA-3a, may be considered the most important, followed by HPA-2, -6, -1, -5, and -4 systems, as a cause of FNAIT, PTP, and PTR based on allele frequency. HPA-4b and HPA-5b role cannot be excluded based on their immunogenicity. A larger-scale study will now be conducted to confirm these hypotheses and to establish an apheresis donor database for the procurement of HPA-matched apheresis platelets for patients with PTR.

## 1. Introduction

Human platelet antigens (HPAs) are polymorphic structures located on platelet membrane glycoproteins. As is the case for ABO and Rh blood groups, platelet antigen frequency varies among ethnic groups. HPA polymorphism leads to the occurrence of human platelet antibodies following alloimmunization against HPA [1]. HPA alloimmunization is responsible for clinically relevant immune-mediated platelet disorders including fetal/neonatal alloimmune thrombocytopenia (FNAIT), posttransfusion purpura (PTP), and platelet transfusion refractoriness (PTR) [2–4]. HPA genotyping in different populations is of importance for predicting the risk of HPA alloimmunization in different ethnic groups [5]. This is essential, as a complement to clinical history, for FNAIT diagnosis and possible future prevention [6–8] and for provision of HPA-matched blood components for patients with PTR [4, 9].

The most extensive studies on HPA gene frequencies have so far been carried out in North America and Western Europe. They have established that, in Caucasians, HPA-1a, located on integrin-β3, is responsible for around 85% of FNAIT cases caused by HPA antibodies [10]. However, estimates of HPA frequency in other populations in the world are still incomplete. In particular, data among oriental populations, including Taiwanese, are still insufficient. In this study, we used sequence-specific primers polymerase chain reaction (SSP-PCR) to screen 998 Taiwanese platelet apheresis donors for 7 clinically significant biallelic human platelet antigens, HPA-1 through -6 and -15. Our goal was (a) to understand which HPA may likely cause FNAIT and (b) to obtain blood donors data to be able to provide HPA-matched platelets for patients with PTR in Taiwan. Since these transfusions may often be required on short notice, it is indeed important to have previously identified suitable donors.

## 2. Material and Methods

### 2.1. Blood Samples and DNA Extraction

998 randomly selected healthy volunteer apheresis platelet donors of six blood centers of the Taiwan Blood Services Foundation (TBSF; Taipei: 325 donors; Hsinchu: 101; Taichung: 97; Tainan: 93; Kaohsiung: 332; and Hualien: 50) were recruited in the study. All the subjects gave informed consent. Blood samples of 3 mL were collected in ethylenediaminetetraacetic acid (EDTA) anticoagulant vacuum tubes and frozen in polypropylene vials at −70°C. DNA was extracted from 0.2 mL of EDTA-treated blood using commercial DNA extraction kit (Texas BioGene Inc., Richardson, TX, USA) and processed according to the manufacturer's instructions. The eluted DNA was diluted to a concentration of 15 ng/**µ**L. 

### 2.2. Sequence-Specific Primer Polymerase Chain Reaction (SSP-PCR)

HPA typing was conducted using SSP-PCR. Samples were tested with the commercial Platelet SSP HPA-1 to -6 and -15 Typing Kit (Texas BioGene Inc., Richardson, TX, USA) according to the manufacturer's instructions. 

### 2.3. Gel Electrophoresis and Gel Interpretation

Amplified DNA products were visualized by UV illumination followed by electrophoresis on 2.0% agarose gel. Results were recorded by ultraviolet photography. For each polymorphic position, one or two possible patterns might be observed: a or b genotype. Samples were classified as HPA a/a, a/b, or b/b genotypes.

### 2.4. HPA Genotyping by Sequence-Based Typing Method (SBT)

To verify the SSP-PCR results, 20 DNA samples were selected for genotyping for HPA-1 to -6 and -15 by SBT. Sequencing primers were designed according to previously published sDNA sequences [11]. DNA samples were amplified, followed by bidirectional sequencing of the amplicons using the ABI BigDye Terminator Cycle Sequencing kit (Applied Biosystems, Foster City, CA, USA) according to the manufacturer's instructions. The sequencing reaction products were electrophoresed using an ABI Prism sequencer (Applied Biosystems). The sequence data were analyzed by SeqScape 2.5 software (Applied Biosystems).

### 2.5. Statistical Analysis

HPA genotype frequencies were estimated by direct counting, and allele frequencies and heterozygosity were calculated by the genetic marker data analysis software (PowerMarker) [12]. Tests for Hardy-Weinberg equilibrium in the population were performed by *χ* ^2^ test. Mismatch probability of different platelet alloantigens, after random platelet concentrate transfusions, was calculated [13] by using the formula 2*ab*(1 − *ab*), where *a* and *b* are the respective HPA gene frequencies at a diallelic focus. The probability of fetomaternal incompatibility between pregnant females and their fetuses regarding different HPA was calculated [14] for assessing the probability of HPA alloantibody development. Comparison between the gene frequencies of six blood centers was done with *χ* ^2^ test using SPSS version 14 (SPSS Inc., Chicago, IL, USA). Where the number of any variable was <5, Fischer's exact test was used to determine the *P* value between two groups.

## 3. Results

### 3.1. HPA Genotyping by SBT

The genotyping results obtained by SSP-PCR were validated using genotyping data by SBT. No discrepancies were observed between the SSP-PCR and SBT typing for HPA-1 to -6 and -15 systems in 20 selected DNA samples, indicating the reliability of the SSP-PCR results.

### 3.2. The Genotype Distributions and Allele Frequency for HPA-1 to -6 and -15

The genotype and allele frequency of HPAs from the 998 apheresis platelet donors are shown in Table 1. HPA-3 and -15 systems exhibited the greatest heterozygosity among all HPA systems. The frequency of a/a, a/b, and b/b genotypes was 29.7%, 52.3%, and 18.0% in the HPA-3 system and 28.4%, 50.7%, 20.9% in the HPA-15 system, respectively. The frequency of HPA-1a/a (99.1%), 2a/a (93.2%), 4a/a (99.5%), 5a/a (97.0%), and 6a/a (95.6%) homozygous genotypes was higher than those of HPA-1a/b (0.9%), 2a/b (6.6%), 4a/b (0.5%), 5a/b (3.0%), and 6a/b (4.3%) heterozygous genotypes. Only two individuals with HPA-2b/b and one with HPA-6b/b had a “rare” genotype, and no b/b homozygote was found in the HPA-1, -4, and -5 systems. There was no significant deviation from the Hardy-Weinberg equilibrium in any of the systems studied in this population. Moreover, the “a” allele was dominant, and the “b” allele was infrequent, in these HPA systems, except for HPA-3 and HPA-15, with frequencies of 55.8%, 44.2% for HPA-3a, HPA-3b and 53.7%, 46.3% for HPA-15a, HPA-15b, respectively. Allele frequency did not differ among blood centers, possibly apart from HPA-6 (not shown).

### 3.3. Estimated Probability of Incompatibility Leading to Risks of FNAIT and PTR

The probability of alloimmunization against the HPA 1 to -6 and -15 systems was estimated as a way to assess the risk of FNAIT and PTR occurrence in Taiwan (Table 2). The probability of fetomaternal mismatch in HPA-1, -2, -4, -5, and -6 was very low. In HPA-3a/a and -3b/b mothers, the risk of mismatch with the fetal antigens during pregnancy was found to be 138/1000 and 109/1000, respectively. It was 133/1000 pregnancies for an HPA-15a/a mother, and 115/1000 pregnancies for an HPA-15b/b mother. The mismatch probability of HPA after random platelet transfusions ranged from 0 to 0.37. The highest risks of incompatibility, in HPA-3 and -15 systems, were 37.16 and 37.76%, respectively, whereas the chances of incompatibility to HPA-1, -2, -4, -5, and -6 were much lower (<7%). These results may help predict the risk of alloimmunization in the patients who will receive random platelet transfusion.

### 3.4. Allele Frequency of Antigens on GPIIIa

GPIIIa is the most polymorphic glycoproteins on human platelet membranes and it bears in particular HPA-1, HPA-4, and HPA-6. Alloantigenic epitopes can induce an alloimmune response. The gene frequency of the allelic forms of these three antigens in Taiwanese is shown in Table 3.

## 4. Discussion

Platelet-specific antibodies may be involved in alloimmune disorders such as FNAIT, PTP, and PTR [15]. HPA genotyping is important for the diagnosis of alloimmune thrombocytopenic syndromes [16]. The distribution frequency of HPA varies among populations worldwide. Data on HPA frequency in Taiwan population have so far been limited and involved small cohorts. We therefore determined the gene frequency of HPA to be close to one thousand apheresis platelet donors from six blood centers located all over the country. To carry out this study, we used SSP-PCR, the recommended method that has proved to be a fast and efficient way to genotype human leukocyte antigen (HLA) and some HPAs [17]. Commercial kits for HPA genotyping have been made available. Here, we used HPA type Platelet SSP HPA-1 to -6 and -15 Typing Kit for HPA genotyping of these 7 HPAs. 

In Caucasian population, HPA-1 is known to be the most important antigen system involved in antiplatelet alloimmunity [15]. In this study, no HPA-1b/1b homozygote was found, which is consistent with previous studies in Taiwan groups [18, 19]. Only nine individuals were found to be HPA-1a/1b heterozygous. These data likely explain the low incidence of FNAIT and PTP due to HPA-1 alloimmunization in Taiwanese. We identified two homozygous b/b genotype individuals for HPA-2, a new observation for Taiwan [18, 19]. Although there is no report of HPA-2 involvement in alloimmune disorders in Taiwan, it may possibly play a more important role than does HPA-1. The genotype frequency of HPA-3a and -3b confirms previous data [18, 19], but immunization against HPA-3a may be more problematic as it is more immunogenic than HPA-3b. No homozygous HPA-4b/b was found in the present study whereas only five heterozygous HPA-4a/b individuals were identified. HPA-5 was considered the second most important immunogenic factor linked with alloimmune syndromes in Caucasians [20]. But in this study, as before [18], no HPA-5b/b was found. The rarity of homozygous HPA-5b/b and the low frequency of HPA-5b may contribute to reducing the probability of platelet incompatibility phenomena involving this antigen in Taiwanese. We found 1 in 998 individuals having HPA-6b/b in the present study while none could be found in 1000 individuals from China [13]. A larger-scale study would be needed to identify whether a difference of HPA-6b/b genotype does exist between Taiwanese and the other Chinese groups. HPA-15, also known as Gov a/b, is considered to be the most relevant HPA after HPA-1 for FNAIT induction [21], especially HPA-15b that is more immunogenic [20]. A report of 605 samples has shown that the Gov a/b platelet alloantigens have an immunogenicity similar to that of HPA-5 alloantigens [22]. In this study, the genotype frequencies of HPA-15a and -15b are 53.71 and 46.29%, respectively, equivalent to that reported by the Chinese study [13]. The frequency of HPA-1a/4a/6b on GPIIIa glycoprotein was about 0.023, which is higher than that (0.013) reported in Chinese Han population [23] and similar to that (0.027) in Japanese [24]. It should be kept in mind that allele frequency is only one of the factors involved in the risks of platelet antigen alloimmunization [25]. Indeed, while the likelihood of antigen incompatibility between mother and fetus is high in HPA-3 and HPA-15 systems, considering the high heterozygosity rate, they may actually be less likely to cause FNAIT compared to other systems. We found that 0.5% of individuals are HPA-4b positive (compared to 0% in Caucasians) and 3.01% are HPA-5b positive; the potential for HPA-4b and HPA-5b to induce alloimmunization cannot be ignored since these alleles are known to be immunogenic [20, 25]. They may therefore be responsible for more FNAIT cases than HPA-3a or HPA-15b.

In conclusion, our data provide information on platelet antigen allele frequency in Taiwan population that can serve as a background (a) to diagnose the cause of FNAIT, PTP, and PTR and (b) to provide HPA-matched apheresis platelet for patients with HPA alloimmune disorders. Large-scale study will now be conducted to confirm these data in order to establish an apheresis donor database for the provision of HPA-matched platelets for transfusion to patients with alloimmune disorders.

## Figures and Tables

**Table 1:** Genotype and allele frequency of HPA in Taiwanese (*n* = 998).

HPA system	Genotype			Allele		Hardy-Weinberg equilibrium	
	a/a (%)	a/b (%)	b/b (%)	a (%)	b (%)	*χ* ^2^	*P *
HPA-1	989 (99.10)	9 (0.90)	0 (0.00)	1987 (99.55)	9 (0.45)	0.0205	0.9898
HPA-2	930 (93.18)	66 (6.61)	2 (0.20)	1926 (96.49)	70 (3.51)	0.5222	0.7702
HPA-3	296 (29.65)	522 (52.30)	180 (18.04)	1114 (55.81)	882 (44.19)	3.6431	0.1618
HPA-4	993 (99.50)	5 (0.50)	0 (0.00)	1991 (99.75)	5 (0.25)	0.0063	0.9969
HPA-5	968 (96.99)	30 (3.01)	0 (0.00)	1966 (98.50)	30 (1.50)	0.2324	0.8903
HPA-6	954 (95.59)	43 (4.31)	1 (0.10)	1951 (97.75)	45 (2.25)	0.5010	0.7784
HPA-15	283 (28.36)	506 (50.70)	209 (20.94)	1072 (53.71)	924 (46.29)	0.3847	0.8250

**Table 2:** Estimate of HPA incompatibility probability for assessing the risk of FNAIT and PTR.

	Frequency major allele (%)	Heterozygosity (%)	Fetomaternal incompatibility		Mismatch platelet transfusion
			Anti-a risk (%)	Anti-b risk (%)	
HPA-1	99.55	0.90	<0.01	0.45	0.89
HPA-2	96.49	6.61	0.12	3.27	6.54
HPA-3	55.81	52.30	10.90	13.76	37.16
HPA-4	99.75	0.50	<0.01	0.25	0.50
HPA-5	98.50	2.96	0.02	1.46	2.92
HPA-6	97.75	4.41	0.05	2.15	4.31
HPA-15	53.71	49.73	11.51	13.35	37.76

**Table 3:** Allele frequencies of three platelet antigens located on GPIIIa in Taiwanese.

HPA-1	HPA-4	HPA-6	Allelic forms	Frequencies
Leu33	Arg143	Arg489	1a/4a/6a	0.9704
Leu33	Arg143	Gln489	1a/4a/6b	0.0225
Pro33	Arg143	Arg489	1b/4a/6a	0.0045
Leu33	Gln143	Arg489	1a/4b/6a	0.0025
